# Spinal Cord Anatomy for Visually Impaired Students: The Development of 3D-Printed Educational Models

**DOI:** 10.59390/001c.163854

**Published:** 2026-07-08

**Authors:** Roberto G. F. Ferreira, Gabrielle G. Garcia, Mariana A.P.C.S.G. Fischer, Daylane M. Maia, Luciana M. Miranda, Karinne C. Lopes, Edgar B.B. Galvão, Cláudio T. Mesquita

**Affiliations:** 1 Morphology Universidade Federal Fluminense

**Keywords:** 3D printing, inclusive education, spinal cord anatomy, visually-impaired students, neuroanatomy

## Abstract

Traditional neuroanatomy education heavily relies on visual perception, creating a significant barrier for visually impaired students. This report describes the development and initial application of tactile 3D-printed models designed to make spinal cord anatomy and neuronal morphology accessible. Using a workflow of digital reconstruction from classical anatomical references followed by Fused Deposition Modeling (FDM) 3D printing, we produced scaled-up, high-relief models of multipolar and bipolar neurons, spinal nerve formation, and distinct spinal cord segments. The models were printed with polylactic acid (PLA) and thermoplastic polyurethane (TPU) to enhance durability and tactile discrimination of delicate structures like nerve rootlets. A pilot evaluation with three blind students demonstrated that the models successfully translated abstract microscopic concepts into concrete, comprehensible objects, enabling the distinction of anatomical features that were previously indistinguishable in cadaveric specimens. Qualitative feedback provided insights into optimal scale, the effectiveness of high-relief modeling for spatial understanding, and the challenges of textural stimulation. Importantly, these tools also proved to be valuable cognitive aids for sighted learners, indicating their universal teaching potential. In summary, these reproducible 3D-printed models are a potential tool for inclusive education, offering an equitable and multisensory learning experience that benefits all students in grasping the complex three-dimensional organization of the human nervous system.

The increasing enrollment of students with disabilities in higher education (Costa & Pieczkowski, 2020) underscores the need for pedagogical innovation. To create equitable learning environments, institutions must critically evaluate traditional methodologies and adopt evidence-based strategies that accommodate diverse sensory and cognitive needs.

In biomedical education, reliance on visual perception poses significant challenges for visually impaired students, particularly in the study of morphology. Neuroanatomy, with its intricate three-dimensional organization and millimeter-scale dimensions of neural pathways and nuclei, challenges spatial conceptualization even for sighted learners (Kramer & Soley, 2002). However, limiting neuroanatomical education to a pre-established “visual” didactic model would unjustly exclude visually impaired students from accessing this knowledge.

In Brazil, inclusive education policies ensure the integration of students with disabilities at all educational levels. Higher education institutions are not only responsible for providing access but also for implementing measures that support student retention and academic success (Brasil, 1996, 2013). At Fluminense Federal University (UFF, Rio de Janeiro, Brazil), direct engagement with medical and psychology students with visual impairments revealed a critical gap in the accessibility of neuroanatomy education. The reliance of traditional academic curricula on visual-based instruction, coupled with a scarcity of sensory-adapted resources, is the primary factor creating an inequitable learning environment and impeding a functional understanding of neuroanatomical systems.

The primary objective of our project was to develop 3D-printed models that replicate conventional anatomical specimens while modifying scale, texture, and surface relief to enable tactile exploration. Three-dimensional printing enables the layer-by-layer fabrication of physical objects from digital designs, ensuring high reproducibility and scalability (Chae et al., 2015; Sander et al., 2017). Prior studies demonstrate its efficacy in inclusive education for visually impaired students (Karbowski, 2020). Despite these advancements, few studies have implemented 3D-printed models in higher education for neuroanatomy instruction (but see Fleming et al., 2020; Giglia et al., 2019; Hadžiomerović et al., 2025; Renna et al., 2024; Ye et al., 2020 for examples). The majority of existing literature relies on manually crafted tactile materials (Diniz & Sita, 2019; Marins, 2020). While manual fabrication reduces costs, additive manufacturing offers superior reproducibility, scalability, and precision, enabling adjustments to scale and replication of intricate details.

At the Health Science & Education Laboratory (Medical Diagnostic Unit, Antônio Pedro University Hospital, UFF/Brazil), we have successfully developed 3D-printed models for undergraduate morphology training (Yahiro et al., 2023) and patient education (Abrantes et al., 2024). Here, we extend these techniques to neuroanatomy.

These models emphasize important anatomical landmarks across spatial planes and provide an essential intermediary step. The tactile exploration of the models was carried out in practical laboratory classes, alongside the same structures present in cadaveric specimens. Initially, the student uses enhanced tactile perception on the three-dimensional models. Then, they use the cadaveric specimens. Finally, the students can compare both, gaining perspective on both enlarged forms and real-scale representations. By developing this technology, we not only broaden educational approaches in the biological sciences for visually impaired students but also provide complementary teaching tools for sighted learners and auxiliary aids to traditional cadaveric specimens (Bialy et al., 2019; DeVeau et al., 2020).

This experience report describes the initial development of 3D-printed neuroanatomical models for blind and low-vision students, focusing on the spinal cord, spinal nerve, and key aspects of neuronal morphology, fundamental elements for understanding the nervous system. We selected the spinal cord as our starting point due to its relative simplicity and evolutionary significance as the precursor to more complex brain structures.

## MATERIALS AND METHODS

### Ethics

This work was approved by the Brazilian National Committee for Ethics in Research.

### Methodological Challenges in Developing 3D Models for Neuroanatomical Education

A primary challenge in this study was converting two-dimensional anatomical structures from conventional atlases (Haines, 2015; Netter, 2019; Splittgerber, 2019) and textbooks (Afifi & Bergman, 2005; Kandel, 2013; Machado, 2014) into accurate, interactive three-dimensional models. Since neuronal morphology is quite complex, we use simpler representations, as in traditional neuroanatomy textbooks, for purely didactic purposes. To address this, we adopted a two-phase workflow: digital reconstruction using specialized 3D-modeling software, followed by additive manufacturing (3D printing).

Neuroanatomical structures, including neurons, spinal cord tracts, and peripheral nerves, were modeled using *ZBrush 2025.3.0* (Maxon)*, v2025 SOLIDWORKS* (Dassault Systèmes) and *Blender 4.4 LTS* (Blender Foundation). For printing, meshes were segmented and sliced in OrcaSlicer 2.3 (RC) and UltiMaker Cura 5.10.1, and exported as binary STL files. The 3D-printed prototypes were produced via Fused Deposition Modeling (FDM), an additive manufacturing technique that layers thermoplastic materials to construct objects, on a Creality Ender-3 V2 FDM printer (0.4-mm nozzle; 220 × 220 × 250 mm build volume; heated glass bed). Materials included polylactic acid (PLA), a biodegradable polyester derived from renewable resources, and thermoplastic polyurethane (TPU), a flexible polymer ideal for delicate structures such as nerve roots and rootlets.

### Development of Neural Cell Models

**Figure 1.**
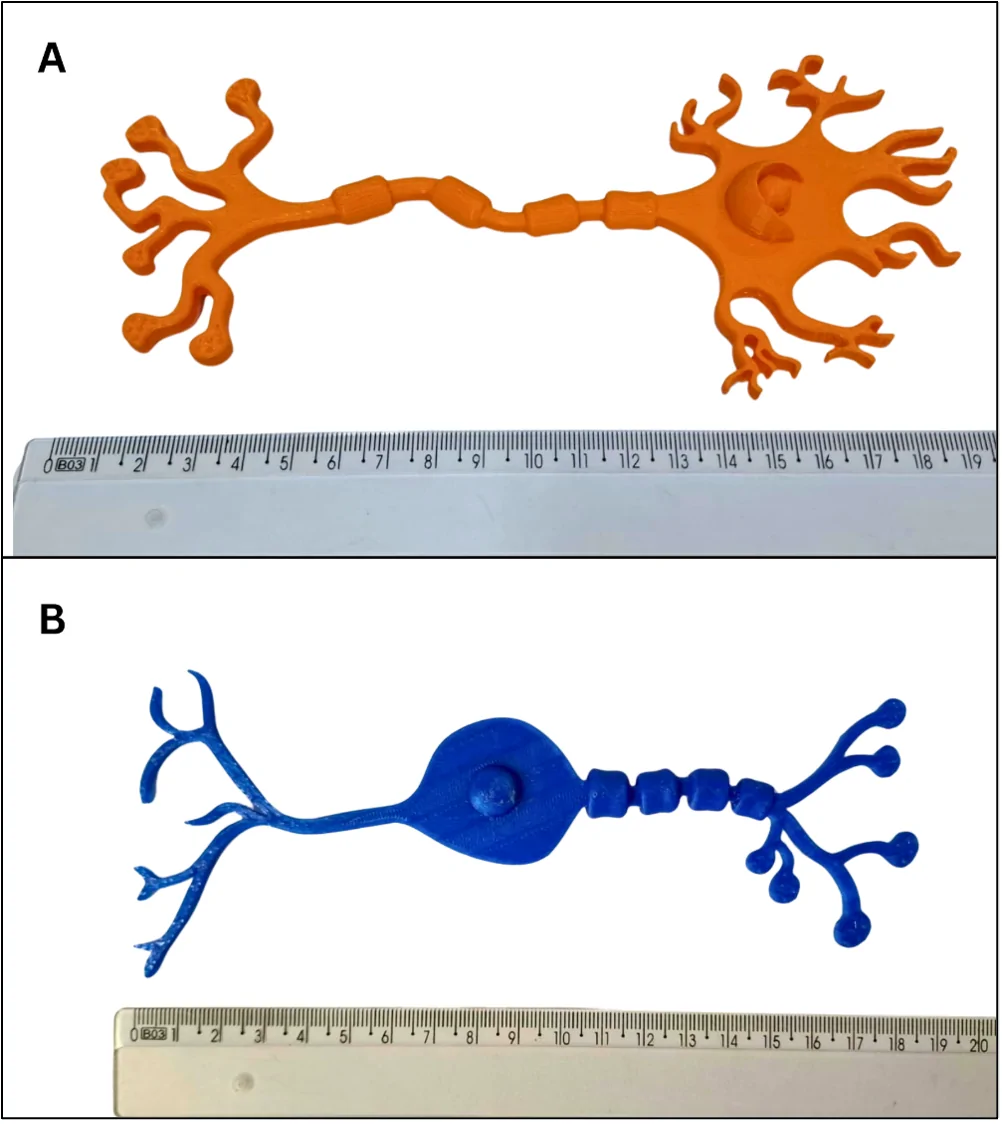
3D-printed models of a multipolar (A) and a bipolar neuron (B), with Schwann cells wrapping their respective axon.

Based on classical histological representations, we modeled a multipolar neuron in SOLIDWORKS with a 7 cm cell body, including six emergent projections representing dendrites and their terminal arborizations. A prominent, rounded nucleus was integrated into the cell body. Extending from the soma, a 13 cm axon was designed with terminal boutons at its distal end. Myelin sheaths, Schwann cells, nodes of Ranvier, and internodes were rendered in relief along the axon to facilitate tactile recognition (Figure 1A). The multipolar neuron was selected as the initial model due to its widespread use in educational materials.

A bipolar neuron model was designed based on the prior multipolar neuron’s layout. The cell body measures 4 cm in length, with two distinct processes extending from opposite poles (Figure 1B). On one side, a single 6.5 cm dendrite emerges, branching into fine terminal arborizations. On the opposite side, an 8.5 cm axon extends distally, featuring characteristic structural components such as Schwann cells, nodes of Ranvier, and internodal segments.

### Development of Spinal Nerve Model and Its Connections to the Spinal Cord

After establishing the structural characteristics of neuronal cells, our next objective focused on enabling visually impaired students to conceptualize spinal nerve formation and its connections to the spinal cord. Traditional anatomical models often fail to convey the fine-scale anatomy of rootlets and nerve roots, which measure just a few millimeters in diameter and are indistinguishable during tactile exploration, particularly when handled with gloves.

To address this, we uniformly scaled all neural structures across the models. For spinal nerves, dimensions were enlarged tenfold relative to natural anatomy. The 3D models were designed around the concept of a spinal cord segment, a defined region responsible for generating a pair of spinal nerves. This approach simultaneously clarifies the spatial organization of nerve pairs and reinforces the theoretical framework of spinal cord segmentation.

**Figure 2.**
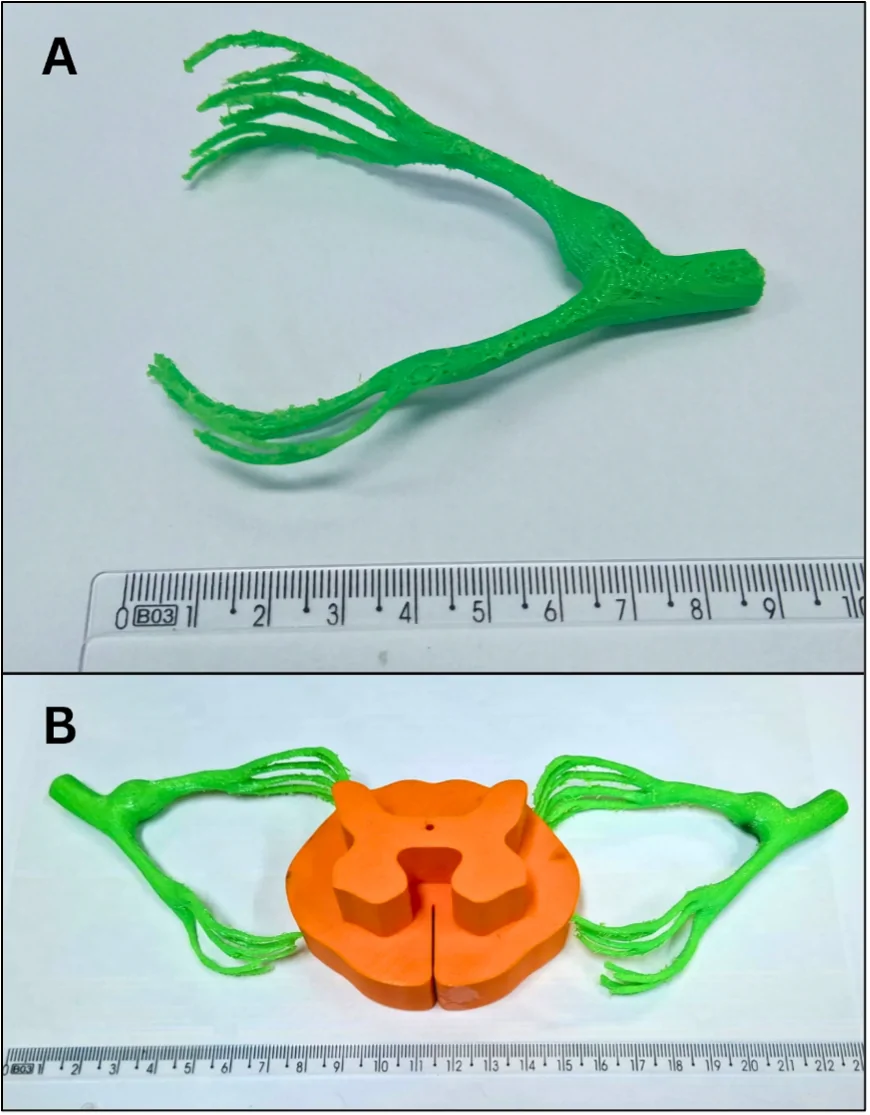
3D-printed models of spinal nerve formation. (A) Isolated model of the spinal nerve formation with dorsal and ventral root components. The dorsal root ganglion is represented by a localized enlargement of the dorsal root. (B) Two spinal nerve models positioned bilaterally alongside a transverse section of the spinal cord. The central H-shaped relief represents the gray matter, whereas the surrounding outer region represents the white matter.

Early prototypes integrated nerve roots directly on the spinal cord surface. Team evaluation indicated that isolating the spinal nerve as an independent structure enhanced tactile recognition of its elements. Consequently, the final models were divided into two components: one featuring detached nerve roots, rootlets, dorsal root ganglion, and spinal nerves (Figure 2A), and another displaying these structures bilaterally to the spinal cord segment (Figure 2B). Direct printing of the models, fixing the root filaments to the medullary segments, did not provide adequate resistance for manipulation, frequently breaking the rootlets. Therefore, we opted for movable models with contact attachment. The spinal cord and spinal nerve parts are 3D-printed as independent structures. During practical classes, both are touched and explored separately by the students. Then, the spinal nerves are anatomically positioned on the spinal cord, with the help of teachers and teaching assistants, and studied together. This allows for a greater three-dimensional understanding of these structures.

The peripheral nerve model was designed in *ZBrush* and 3D-printed in thermoplastic polyurethane (TPU). TPU’s flexibility and durability allowed the fabrication of delicate neural structures while ensuring resilience during tactile interaction, critical for repeated handling by students.

This two-stage pedagogical strategy begins with tactile exploration of the isolated peripheral nerve model to solidify understanding of its components. Students then progress to the integrated spinal cord model, where rootlets are affixed bilaterally in relief. The spinal cord segment incorporates tactile relief differentiating gray and white matter, enabling learners to spatially contextualize nerve roots and rootlets relative to these regions and observe bilateral symmetry of spinal nerves. This progression reinforces both tactile and conceptual understanding of spinal cord segmentation.

### Three-Dimensional Structural Modeling of Spinal Cord Gray Matter

Traditional study of the spinal cord relies on the spatial distinction between white and gray matter. In dissected specimens, the lighter-colored white matter, located peripherally, contrasts with the darker gray matter in the center. While these distinctions are readily observable in transverse sections by sighted students, they are not accessible through tactile perception by visually impaired students.

To create a more inclusive, multisensory learning experience, we developed models in which the gray matter, shaped like the letter “H”, is presented in raised relief, extending several centimeters from its original plane. The white matter was removed from the upper section and reintroduced at the inferior portion of the model as a peripheral boundary. This approach allows students to perceive the spatial morphological characteristics of gray matter as a consolidated structure of neuronal cell bodies centered along the neural axis and extending throughout the spinal cord. It also clarifies the position of the white matter that surrounds the “H” shape and the proportional relationship between the two (Figure 2B).

The three-dimensional projection further enables tactile identification of the anterior, posterior, and lateral columns, as well as the intermediate gray matter and the central canal. These components of the spinal cord’s gray matter are formed by distinct sets of neuronal cell bodies and are important for understanding the regions responsible for voluntary movements, autonomic movements, and sensory functions. We also prioritize the location of the spinal funiculi, the pathways through which the various modalities of motor, sensory, and associative bodily impulses pass. To facilitate tactile understanding of the columns and funiculi of the spinal cord, we have enlarged the anatomical dimensions of the original human spinal cord by approximately fifteen times.

**Figure 3.**
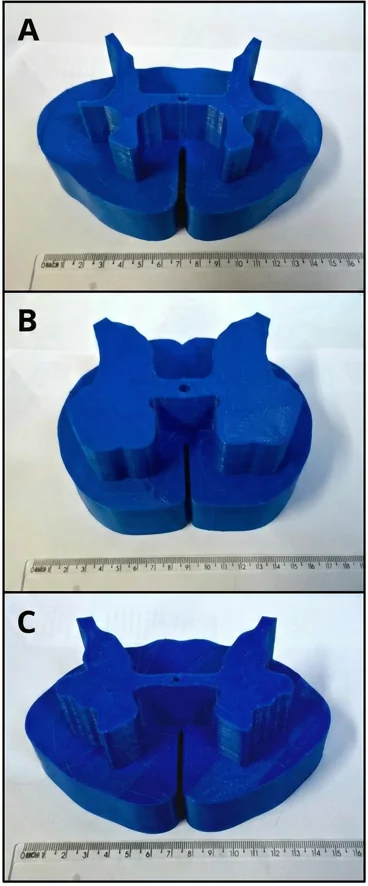
Morphometry of thoracic (A), lumbar (B) and cervical (C) spinal cord segments. The central H-shaped relief represents the gray matter, while the surrounding region represents the white matter organized into posterior, lateral, and anterior funiculi. Compared with the thoracic segment, the lumbar segment shows a greater proportion of gray matter, whereas the cervical segment shows a greater proportion of white matter.

We produced models emphasizing the specific characteristics of the cervical, thoracic, and lumbosacral regions. In this way, it was possible to highlight the morphological characteristics of each spinal cord level. This method not only emphasizes the anatomical differences within the gray matter and the varying proportions of white matter across segments (Figure 3), but it also retains the tactile delineation of the spinal cord funiculi, which is maintained at the base of each model.

### Development of Structural Models of White Matter and Spinal Cord Pathways

**Figure 4.**
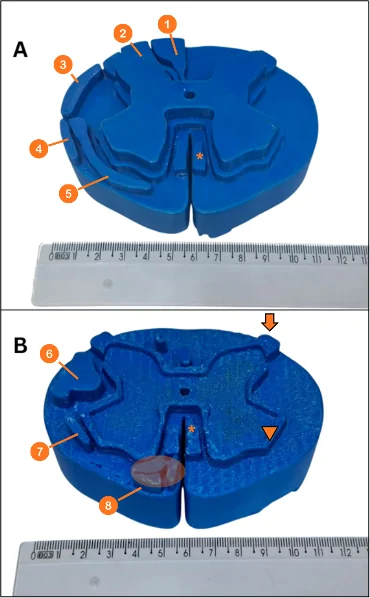
Dual-sided cervical spinal cord model showing ascending and descending tracts. One surface of the model represents the sensory pathways (A), displaying the gracile fasciculus (1), cuneate fasciculus (2), posterior spinocerebellar tract (3), anterior spinocerebellar tract (4), and anterior and lateral spinothalamic tracts (5). The opposite surface represents the motor pathways (B), displaying a combined representation of the lateral corticospinal and rubrospinal tracts (6), the medullary reticulospinal tract (7), pontine reticulospinal tract, tectospinal tract, and anterior corticospinal tract (8). On both surfaces, the medial longitudinal fasciculi are present bilaterally and indicated by asterisks, propriospinal fibers by the arrowhead, and the dorsolateral fasciculus by the arrow.

To represent the ascending and descending tracts within the funiculi of the spinal cord, we maintained the design of previous models, presenting the gray matter in high relief and the white matter in low relief relative to the base plane. The neural pathways were then represented in an intermediate relief plane, lower than the gray matter, and higher than the dividing plane of the funiculi (Figure 4 A and B). This configuration allows visually impaired students to discern, by touch, the spatial relationships within the spinal cord and locate the pathways in relation to the funiculi and the H-shaped gray matter.

A cervical spinal cord segment was selected due to its inclusion of major spinal tracts and higher proportion of white matter. A single dual-sided tactile model was developed to represent sensory and motor pathways on opposite surfaces of the same piece. One surface, shown in Figure 4A, displays the anterior and lateral spinothalamic tracts, anterior and posterior spinocerebellar tracts, gracile and cuneate fasciculi, and the propriospinal fibers. The opposite surface, shown in Figure 4B, displays the motor pathways, including the lateral and anterior corticospinal tracts, as well as the rubrospinal, reticulospinal, and tectospinal pathways. To enhance tactile discrimination, the pathways were arranged asymmetrically, with tracts concentrated in one hemicord on each surface, while the contralateral hemicord either lacks tracts or displays only associative fibers adjacent to the gray matter, clarifying their spatial relationship to the funiculi.

**Figure 5.**
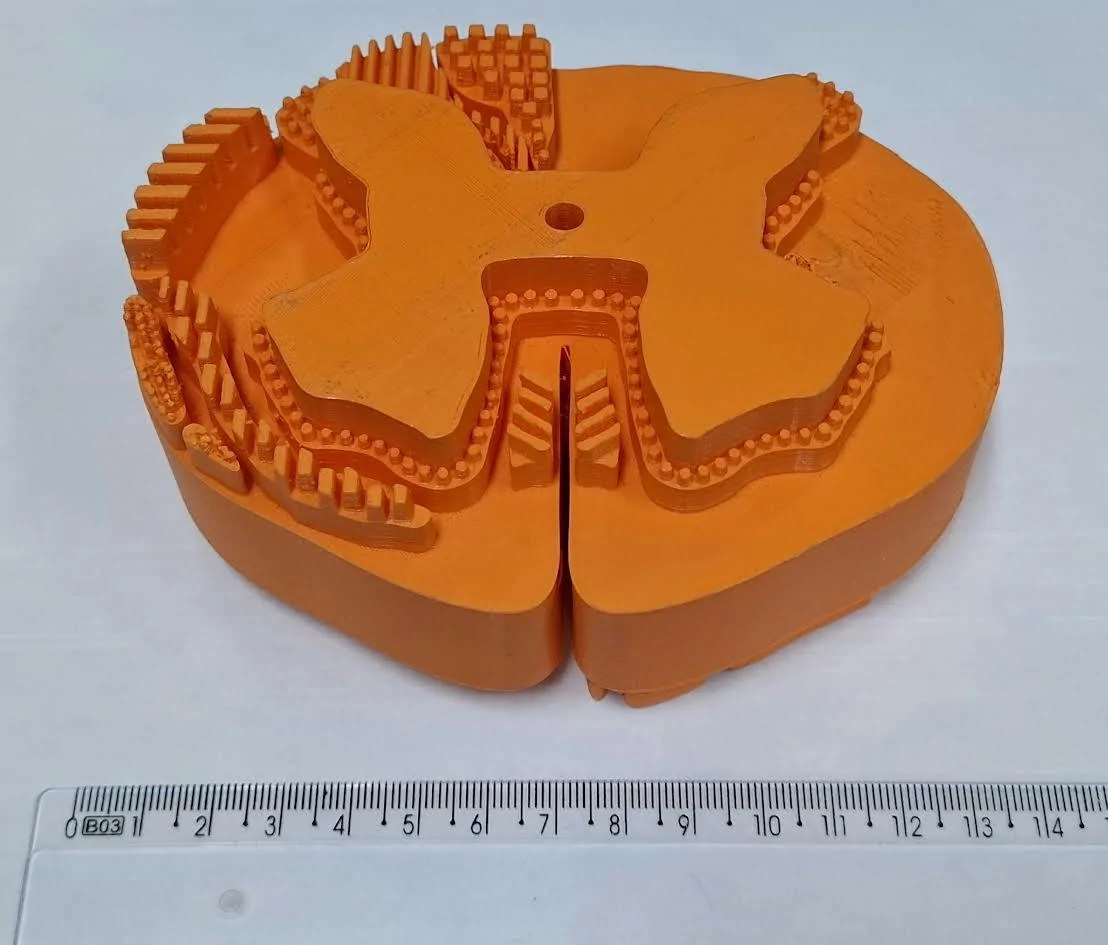
Spatial Representation of Spinal Pathways using different textures. Striated patterns with different orientations represent the cuneate fasciculus, posterior spinocerebellar tract, and anterior and lateral spinothalamic tracts. Small dots represent the anterior spinocerebellar tract. The medial longitudinal fasciculus is represented bilaterally by striated textures, whereas the propriospinal fibers and dorsolateral fasciculus are represented bilaterally by a large, dotted texture.

A subsequent prototype, based on the previous dual-sided model of ascending and descending spinal pathways, was developed to improve tactile discrimination through differentiated surface textures applied to groups of neural pathways (Figure 5). The anatomical distribution of the tracts was preserved, while the intermediate relief planes were redesigned with distinct tactile patterns, including hatching, small raised squares, and stippling. This strategy was intended to help users distinguish adjacent pathways by touch while maintaining the spatial and topographical relationships of the tracts within the white matter.

### Model Availability

The models described in this article are freely available for download under a CC BY 4.0 license at https://3d.nih.gov/users/gabrielle-gomes. They are provided in standard 3D file formats suitable for visualization, further editing in compatible modeling software, and preparation for 3D printing. For better results we recommend using PLA as the main filament and using the same size parameters presented in the figures above. The corresponding author makes their email available to assist educators or clarify any doubts regarding the 3D printing process.

## RESULTS

To evaluate the pedagogical utility of the 3D models, a pilot, hands-on session was conducted with three visually impaired students: one psychology and two medical students. The participants had already completed the standard neuroanatomy course with their peers. This course, led by the discipline’s professors and teaching assistants, was based on theoretical classes and practical laboratory work with cadaveric specimens. In the pilot project, students interacted with 3D-printed models for the necessary time, with the assistance of a neuroanatomy professor and teaching assistants. This setup allowed for a comparison between the students’ initial experiences with cadaveric specimens and the new experience with the corresponding three-dimensional models. Student feedback was collected verbally at the end of the pilot project.

After reviewing neuronal cell features, the session focused on the formation of spinal nerves and their continuity with the spinal cord. In prior practical sessions, the students indicated that latex gloves and the small caliber of the anatomical elements made the dorsal and ventral rootlets, their convergence into nerve roots, and the dorsal root ganglion feel like a single, undifferentiated structure. In contrast, the 3D-printed models enabled logical, stepwise tactile exploration. The participants were able to begin by identifying the discrete rootlets emerging from the cord surface, then trace their convergence into clearly distinguishable dorsal and ventral roots. The students emphasized that this sequence clarified component boundaries that had previously been indistinguishable.

To assess the role of scale in tactile perception, models of the spinal cord at sacral, thoracic, and cervical levels were printed in various sizes. The participants’ feedback revealed a direct correlation between model size and the ability to discriminate between gray and white matter. Models with approximate dimensions of 15 cm in lateral diameter x 10 cm in anteroposterior diameter x 7 cm in height were identified as ideal for this purpose (Figure 3). This finding suggests that proportional enlargement, when combined with high-relief modeling of internal features, is a decisive factor in translating theoretical knowledge into a spatially anchored understanding for learners with visual impairments.

In their verbal comments to the instructors, the students reported that without the models, the structures were only imagined. To quote some of their verbatim: “Before these models, the nerve roots and ganglia were just theoretical concepts I had to memorize; now, I can actually feel where one structure ends and another begins” and “It made me finally understand how the white and gray matter change according to the cross-section of the spinal cord.” With the manipulation of the models, it was possible to understand, for the first time, the different anatomical structures and their spatial relationships.

## DISCUSSION

A primary challenge in neuroanatomy is the abstract nature of microscopic structures. Qualitative feedback from the participants indicated that the models provided unprecedented tactile access to these concepts. For instance, the histological organization of a multipolar neuron, typically conveyed only through two-dimensional atlas images, became comprehensible for the first time through direct manipulation of the enlarged 3D model and its fine substructures. The model effectively translated an abstract, visual concept into a concrete, physical object.

Further exploration focused on using textural variation to encode different white matter tracts. While distinct textures enhanced the identification of individual pathways by providing a unique sensory “signature” applying multiple textures to a single, complex model resulted in tactile overstimulation, which the participants described as overwhelming. This feedback suggests that a more effective design principle may involve modularity. Presenting pathways as detachable, “plug-in” components, each with a distinct texture, would allow learners to explore tracts individually before integrating them into the complete structure.

Importantly, the same models demonstrated utility for sighted learners (Costa & Pieczkowski, 2020), which indicates a universal teaching potential of these tools (Fleming et al., 2020; Giglia et al., 2019; Hadžiomerović et al., 2025; Renna et al., 2024; Ye et al., 2020). Informal oral feedback from visually impaired students during pilot sessions indicated that the models served as effective cognitive aids. Specifically, they improved the spatial comprehension of long spinal pathways and the three-dimensional topography of gray matter, complementing the study of traditional specimens.

It should be noted that the 3D models described here are already being used with our students in practical neuroanatomy classes as an auxiliary tool for teaching cadaveric models. Although this research is in its early stages, the models have already been applied in our laboratories to 80 psychology students and 160 medical students, with promising results from sight students.

The primary limitations of this pilot study are small sample size and qualitative nature. While the outcomes are encouraging and have informed successive design refinements, they cannot be generalized. To move beyond qualitative impressions, we are developing a prospective evaluation plan to quantify effectiveness in future cohorts. This will allow us to assess learning gains attributable to the models and to optimize design parameters such as scale, relief depth, and textural coding.

As the models are used, new demands from students and the course arise, requiring new solutions. Anatomical features of the spinal cord, such as the crossing of sensory and motor pathways, were not demonstrated at the time of writing this text. Similarly, the positioning of the spinal nuclei that form the spinal columns was not indicated in the current models. However, new models are being made to meet this demand. An important observation, made by visually impaired students, is the need to limit tactile information in each model, so that the learning objective is clear in each model – for example, one model emphasizes spinal pathways, another emphasizes gray matter morphology, and another the spinal funiculi.

Finally, by publicly sharing these models and our initial experience, we hope that other educators can adopt our approach, and perhaps even assist us in the development of future models.

In summary, the translation of complex, microscopic neuroanatomical features into scalable, tactile 3D-printed models provides a highly impactful, multisensory, and inclusive learning experience. These tools bridge the gap between abstract theory and concrete understanding for visually impaired learners, while also serving as valuable spatial references for sighted students, thereby fostering a universally accessible educational environment.

### Address correspondence to:

Roberto Godofredo Fabri Ferreira, Department of Morphology, Fluminense Federal University (UFF), 57 Alameda Barros Terra, Niteroi, Rio de Janeiro, Brazil, 24020-150. Email: robertofabri@id.uff.br
